# Clinical Aspects of Scoliosis

**Published:** 1895-06-08

**Authors:** A. H. Tubby

**Affiliations:** Assistant Surgeon to the Westminster Hospital; Surgeon to the National Orthopædic Hospital; Surgeon to Out-patients Evelina Hospital for Sick Children


### MEDICAL PROGRESS AND HOSPITAL CLINICS.

[*The Editor will be glad to receive offers of co-operation and contributions from members of the profession. All letters should be addressed to* THE EDITOR, THE LODGE, PORCHESTER SQUARE, LONDON, W.]

### CLINICAL ASPECTS OF SCOLIOSIS.

By A. H. TUBBY, M.S.Lond., F.R.C.S.Eng., Assistant Surgeon to the Westminster Hospital; Surgeon to the National Orthopædic Hospital; Surgeon to Out-patients Evelina Hospital for Sick Children.

(*Continued from page* 148.)

To continue the clinical study of scoliosis:—

2. **Cases are seen with Two Equal Curves Present.**—As a rule, the curves have their convexity to the right in the dorsal, and to the left in the lumbar or dorso-lumbar region. Rarely, however, the two curves are situated in the cervical and dorsal portions of the column. In either of the above conditions the characteristic S-shaped curve appears, one limb of which is primary, and the second is compensatory. As to which is primary, it is not essential to determine on mere pathological grounds, except in so far as the question of right treatment is concerned; but one may usually take it, that after osseous changes have occurred it is the most important curve which has become fixed, which therefore demands the most attention. It may happen that in some cases the reverse condition of S-shaped curvature is seen, and then the dorsal convexity is to the left and the lumbar convexity to the right. It is necessary, therefore, in depicting the results of such malformations to remember that the distortion will be the reverse of that of the more common deformity described below. That the dorsal convexity to the right is more frequent admits of no dispute. The statistics quoted by Mr. Adams fully support the generally received opinion. Of 569 cases in the dorsal region, 470 were convex to the right and 99 to the left.[1] As to the reason of this preponderance of right-sided dorsal curves, I believe it is due to excessive use of the right arm in faulty positions, occupations, and employments, these being such as elevate the right shoulder and depress the left. Such occupations are clerking among men, painting and sewing in women, and in school-children the very absurd position they are forced to assume in learning to write the "Italian hand." The desk is often too low, and the child is compelled to stoop over it, with the right arm raised and rigid to ensure correct and fine up-strokes, while the left arm is depressed to keep the copybook firm. Such a case of incipient curvature is the following.

Case.—H. T., aged 14, complained of pain in the mid-scapular region, especially severe after school hours. His mother brought the boy to me in July, 1894, because she had noticed the right shoulder growing out. I ascertained that for the last two years he had been sitting at a desk too low for him, and compelled to write in the faulty manner just mentioned.

On examination, I found a very tender place extending over the spines of the third and fourth dorsal vertebræ, so tender on pressure as to make one consider the possibility of the case being one of commencing caries. The legs were equal in length, and the boy was, on the whole, well developed for his age. There was no rigidity of the back on applying the palm pressure test during flexion and extension, but there were two curves present, the upper convex to the right, extending from the second to the ninth dorsal vertebra, and the lower from the tenth dorsal into the lumbar region, with the convexity to the left. The natural curve backwards in the lower dorsal region was somewhat lost., *i.e.*, there was some flattening present, and the left side of the chest projected. He was advised to cease attending school, and to employ a combination of rest and exercises. In October, 1894, the back was nearly straight, the shoulders were almost at the same level, and he had entirely lost the pain. In January, 1895, his figure was completely restored.

In some cases, however, the scoliosis is due to excessive shortening of one leg, or to the habit of standing on one leg. In such instances the lumbar curve is the first to appear. But as a rule it may be said that the higher in the dorsal region is the first curve, the earlier in life has it appeared.

The deformity in the neighbouring parts is seen to be closely analogous to that already described in curves mainly simple. Thus the right scapula is unduly raised, prominent and altered in direction; the right shoulder is higher, the ribs stand out even more than when the curve is of greater length, and the hollow of the right flank is also more marked, but the left hip is in these cases of equal curves always the more prominent. The cavity of the chest is more oblique from side to side owing to the greater projection backwards of the ribs on the convex side; in females the right breast has receded considerably, and the left is unduly prominent. The greater the lateral deviation and rotation and the more closely is it limited to the dorsal region the more disastrous are the effects on the shape of the thorax. Another effect is diminution in height of the spinal column, the result of rotation of the vertebræ associated with lateral flexion.

3. **Three or More Curves Present.**—In some cases three and even four curves are seen, and are variously distributed in the spine. In that form of scoliosis in which there is one large curve present there are sometimes two smaller compensatory ones, but this condition is distinct from that now under consideration. The curves in the latter are often of equal length, and it is difficult to say which is the primary curve. The chief point on which a decision must rest is that the first curve is the most fixed when the patient is suspended.

When three or even four curves are present the accompanying distortion in the chest is less, inasmuch as in each curve fewer vertebræ are affected; but it would only lead to confusion to enumerate all the resulting deformities, even if it were possible to do so accurately. Suffice it to say that on the convex side of the curve the changes will be in principle those enumerated above, and that the distortion effects will be most marked in the chest when the curves are mainly dorsal. On the concave side the reverse conditions will naturally obtain.

4. **Scoliosis Associated with Posterior Projection of Some of the Spinous Processes.**—This condition is seen when a back presents two curves nearly equal, and the projection is found at the spot where the upper and lower curves intersect. The mechanism at work producing the projection is, I take it, as follows: The prominent vertebræ are acted upon by two parallel forces, one above and the other below, *i.e.*, "a couple" of equal and opposite forces occurs. The vertebræ are, therefore, maintained in the middle line, but undergo considerable oblique pressure from above downwards and from below upwards. In some instances they are forced forwards into the cavities of the thorax and abdomen, but more often, in order to escape undue pressure, they yield backwards, hence the prominence of the spinal processes. A certain amount of projection is also due to the mere development or wasting of muscles at the meeting-point of the curves.

The chief interest of this class of case lies in these facts. Projection of the spinous processes is a constant accompaniment of Pott's disease, and lateral deviation an occasional feature; lateral deviation and rotation of the vertebræ are the distinguishing features of scoliosis, and projection of some spinous processes an occasional occurrence. It therefore happens that at first sight some difficulty arises in the diagnosis, but the rigidity and fixity of the affected spinous processes are definite and diagnostic features of Pott's disease.

5. **Scoliosis with Obliteration or Reversion of the Natural Antero-Posterior Curves of the Spine.**—Cases of this description present either the less degree of flattening of the back, or the greater one of reversion of the natural kyphosis in the dorsal region, with flattening or some projection in the lumbar region. It is evident that such an appearance in the mid-region of the spine is due to two causes.

*a.* Considerable rotation of the vertebral bodies around a horizontal axis situated near the apices of the spinous processes, and consequent sinking of the bodies of the vertebræ into the cavity of the chest, where they are deficient in support.

*b.* The posterior projection of the ribs on the convex side.

The flattened lumbar region is due to a compensatory backward pushing of those vertebræ; and such displacement may occur that two or three upper lumbar vertebræ near the intersection of the curves actually form a distinct "bow" in the outline of the spinous processes.

The clinical importance of recognising this group is considerable, inasmuch as the most troublesome factor of lateral curvature of the spine, excessive rotation, is here present, and the prognosis is distinctly unfavourable. It may be laid down as a certain rule that in such instances the external curvature is no measure of the extent of the internal curvature.

[1] Adams' "Curvature of the Spine," 2nd edition, p. 160.

